# *Enterocytozoon bieneusi* Microsporidiosis in Stem Cell Transplant Recipients Treated with Fumagillin^1^

**DOI:** 10.3201/eid2306.161825

**Published:** 2017-06

**Authors:** Iryna Bukreyeva, Adela Angoulvant, Inès Bendib, Jean-Charles Gagnard, Jean-Henri Bourhis, Sylvie Dargère, Julie Bonhomme, Marc Thellier, Bertrand Gachot, Benjamin Wyplosz

**Affiliations:** Centre Hospitalier Universitaire de Bicêtre, Le Kremlin-Bicêtre, France (I. Bukreyeva, A. Angoulvant, I. Bendib, J.-C. Gagnard, B. Wyplosz);; Université Paris-Sud, Le Moulon, France (A. Angoulvant);; Institut Gustave Roussy, Villejuif, France (J.-H. Bourhis, B. Gachot);; Centre Hospitalier Universitaire de Caen, Caen, France (S. Dargère, J. Bonhomme);; Centre Hospitalier Universitaire Pitié-Salpêtrière, Paris, France (M. Thellier);; Université Pierre et Marie Curie, Paris (M. Thellier)

**Keywords:** Enterocytozoon bieneusi, protozoa, microsporidia, microsporidiosis, parasites, obligate intracellular parasite, transplantation, allogeneic, hematopoietic stem cell transplant recipients, immunocompromised patients, antiprotozoal drugs, fumagillin

## Abstract

*Enterocytozoon bieneusi* microsporidiosis is an emerging disease in immunocompromised patients. We report 2 cases of this disease in allogeneic hematopoietic stem cell transplant patients successfully treated with fumagillin. Thrombocytopenia occurred but without major adverse events. Modifications of immunosuppression could be avoided when *E. bieneusi* is rapidly identified and fumagillin therapy is started promptly.

*Enterocytozoon bieneusi*, the most common cause of microsporidiosis in humans (*1*), causes chronic diarrhea and severe wasting syndrome in immunocompromised patients (*2*). In 2002, oral fumagillin was established as an effective treatment for *E. bieneusi* microsporidiosis in HIV-infected and solid organ transplant (SOT) patients (*3*). In contrast to previous treatments that did not result in parasitologic clearance or clinical remission, fumagillin showed a cure rate of 100%, even for severely immunocompromised patients (*2*,*4*–*10*).

Thrombocytopenia is the main adverse event of fumagillin therapy, occurring in up to 33% of patients (*3*) and raising concerns about fumagillin use in patients with hematologic disorders. We report 2 cases of *E. bieneusi* microsporidiosis in allogeneic hematopoietic stem cell transplant (HSCT) recipients who were treated with fumagillin and experienced thrombocytopenia.

Patient 1 was a 50-year-old woman admitted to Centre Hospitalier Universitaire de Caen (Caen, France) after profuse watery diarrhea and abdominal discomfort for 3 weeks. She had not traveled abroad. Three years earlier, she received a genoidentical allogeneic HSCT for myeloid leukemia. She recently had cutaneous chronic graft-versus-host disease. Her immunosuppression regimen used was prednisone and mycophenolate mofetil.

At admission, the patient was dehydrated and had a weight loss of 3 kg. Laboratory analyses showed lymphocytopenia (960 lymphocytes/mm^3^), reference neutrophil (5,100 cells/mm^3^) and platelet (408,000 platelets/mm^3^) counts, and a C-reactive protein level <5 mg/L.

Results of fecal sample analyses were negative for pathogenic bacteria and viruses. Microscopic examination of fecal smears stained with Weber-Green–modified trichrome showed microsporidia. *E. bieneusi* was identified by using monoclonal antibodies (IFA-MAbs; Bordier Affinity Products, Crissier, Switzerland).

The mycophenolate mofetil dose was reduced by 50% for 8 days but no benefit was shown. Resolution of symptoms occurred <5 days after initiating fumagillin therapy (60 mg/d for 14 d); fecal smears were negative for microsporidia on day 9, and transient thrombocytopenia (131,000 platelets/mm^3^) was observed on day 18 (Figure). Fecal smears remained negative for *E. bieneusi* during the 6-month follow-up. No clinical relapse occurred.

**Figure:**
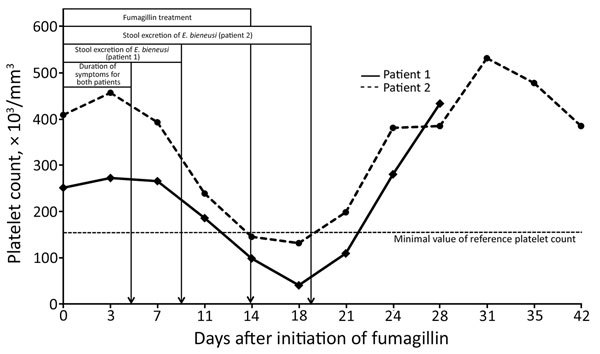
Platelet counts and clinical and parasitologic characteristics during fumagillin therapy and a 1-month follow-up of 2 allogeneic hematopoietic stem cell recipients with *Enterocytozoon bieneusi* microsporidiosis.

Patient 2 was a 42-year-old man referred to Centre Hospitalier Universitaire de Bicêtre (Le Kremlin-Bicêtre, France) after profuse acute diarrhea for 2 weeks and a weight loss of 10 kg. He had not traveled abroad. Four years earlier, he received a genoidentical allogeneic HSCT for acute leukemia. During the follow-up period, he was given a diagnosis of chronic graft-versus-host disease. He was given extracorporeal phototherapy with mycophenolate mofetil, sirolimus, and prednisolone.

At admission, the patient was afebrile and dehydrated. Blood analyses showed severe lymphocytopenia (400 lymphocytes/mm^3^), reference neutrophil (4,680 cells/mm^3^) and platelet (251,000 platelets/mm^3^) counts, and a C-reactive protein level <5 mg/L. Results of microbiological analyses of fecal samples were negative for viruses and pathogenic bacteria. Microscopic examination of fecal smears stained with Weber-Green–modified trichrome showed microsporidia. *E. bieneusi* was identified by using monoclonal antibodies.

The patient was treated with fumagillin (60 mg/d for 14 d) (Figure). Immunosuppressive therapy was not modified. Clinical symptoms resolved within 5 days. Platelet counts progressively decreased. Fumagillin was withdrawn on day 14, but thrombocytopenia worsened (40,000 platelets/mm^3^) by day 18. However, the patient spontaneously recovered in 10 days without any bleeding. No relapses were observed. Microsporidia were not detected in fecal samples during the 6-month follow-up.

*E. bieneusi* is an emerging pathogen in immunocompromised patients (*1*). Increasing numbers of cases have been reported in SOT patients. We report 2 cases of *E. bieneusi* microsporidiosis in allogeneic HSCT recipients who were treated with fumagillin without modifying the immunosuppressive regimen for 1 recipient. In France, fumagillin can be obtained from the French National Agency for Medicines and Health Products Safety (Saint-Denis, France) after an individual patient expanded-access request is submitted.

Clinical and microbiological responses for the 2 case-patients were similar to those reported for other immunocompromised patients (*3*). No relapses were observed for 4 HIV-infected patients whose CD4 cell counts remained low, or for 2 SOT recipients who did not receive tapering immunosuppressive therapy (*3*). In other studies, 15 (70%) of 21 patients treated with fumagillin were cured without modifying immunosuppression regimens (*2*,*6*,*10*); for 6 other patients, immunosuppressive therapy was tapered (n = 4) or withdrawn (n = 2), but reasons for modifying immunosuppression were not specified. For 1 of our patients, the mycophenolate mofetil dose was reduced by 50% to decrease the immunosuppression level. However, no benefit was observed. In contrast, fumagillin led to clinical remission within 5 days.

We observed thrombocytopenia (platelet count <40,000/mm^3^) in both patients but no evidence of bleeding. In other non-AIDS patients, thrombocytopenia was reported in 11 (52%) SOT patients receiving fumagillin, including 4 patients with severely low platelet counts (<25,000/mm^3^) (*1*,*4*,*6*). For these patients, including those we report, thrombocytopenia occurred during the second week of treatment; a minimum value was observed a few days after completing fumagillin therapy. Spontaneous recovery occurred within 2 weeks. Bleeding, hematoma, or requirements for platelet transfusions were not reported. For both patients we report, microsporidia were not detected in fecal samples of both patients during the 6-month follow-up.

In conclusion, fumagillin was highly efficient in curing *E. bieneusi* microsporidiosis in 2 allogeneic HSCT recipients. Thrombocytopenia occurred but without major adverse events. Modifications to immunosuppression could be avoided when *E. bieneusi* is rapidly identified and fumagillin therapy is started promptly.
